# Clinical utility of semi–automated EEG electric source imaging of interictal discharges in presurgical evaluation and surgical treatment decision making

**DOI:** 10.3389/fneur.2025.1598265

**Published:** 2025-09-03

**Authors:** Jelena Hyppönen, Henri Eronen, Anni Saarela, Päivi Koskenkorva, Esa Mervaala, Reetta Kälviäinen, Leena Jutila

**Affiliations:** ^1^Department of Clinical Neurophysiology, Kuopio Epilepsy Center, Kuopio University Hospital, Full Member of ERN EpiCARE, Kuopio, Finland; ^2^Faculty of Health Sciences, School of Medicine, Institute of Clinical Medicine, University of Eastern Finland, Kuopio, Finland; ^3^Department of Neurology, Kuopio Epilepsy Center, Neurocenter, Kuopio University Hospital, Full Member of ERN EpiCARE, Kuopio, Finland; ^4^Department of Child Neurology, Kuopio Epilepsy Center, Neurocenter, Kuopio University Hospital, Full Member of ERN EpiCARE, Kuopio, Finland; ^5^Department of Clinical Radiology, Diagnostic Imaging Center, Kuopio University Hospital, Full Member of ERN EpiCARE, Kuopio, Finland

**Keywords:** electrical source imaging, ESI, epilepsy surgery, decision-making, clinical utility

## Abstract

**Purpose:**

We aimed to evaluate the clinical utility of the semi-automated EEG source imaging (ESI) analysis results during presurgical workup in epilepsy surgery candidates.

**Methods:**

Forty consecutive presurgical patients with focal epilepsy, evaluated during 2021–2022, with a mean age of 35.0 years (range, 2–59), were included. The long-term video-EEG recordings were analyzed using Epilog PreOp (Clouds of Care, Ghent, Belgium). Automatically detected spike clusters were evaluated, and source localization at the half-rising phase of the averaged spike peak was used for ESI localization at the lobar level. The evaluation by the multidisciplinary epilepsy surgery team (MDT), including clinical and multimodal presurgical evaluation data, was used to assess the clinical utility of ESI results in the decision-making process.

**Results:**

Twenty of 40 patients were classified as non-lesional based on magnetic resonance imaging (MRI), and 22 of 36 had focal PET hypometabolism. In automated ESI analyses, no epileptic spikes were detected in four patients (10%). The largest spike clusters were localized to the temporal (58%), frontal (23%), and occipital lobes (3%) or the insula (8%). ESI results had an impact on the suggested treatment choice in 45% of cases and did not significantly affect clinical decision-making in 35% of cases. In patients who underwent resective surgery or had MRI lesions in the temporal lobe, ESI findings were more likely to be consistent with other findings and to complement clinical decisions (Pearson chi-square *p* = 0.016 and 0.027).

**Conclusion:**

This study demonstrates that commercial automated spike detection analyses and source localization, when reviewed by an experienced MDT, can aid clinical decision-making in about 40% of patients, especially for patients in whom resective epilepsy surgery is a feasible option.

## Introduction

Epilepsy, a brain disorder characterized by recurrent and unprovoked seizures, poses a significant burden to affected individuals’ quality of life and overall well-being. For a subset of patients with pharmacologically resistant focal epilepsy, surgery remains an important intervention that could lead to seizure freedom and improved quality of life (1). The success of epilepsy surgery is contingent upon the precise localization of the epileptogenic zone (EZ) (2).

Electroencephalography (EEG) source imaging (ESI) stands at the forefront of modern neuroimaging techniques, offering a model-based approach for pinpointing and visualizing the sources responsible for the electric potentials detected in EEG recordings (3, 4). Primarily utilized in the presurgical evaluation of patients with refractory focal epilepsy, ESI shows promise for enhancing our understanding and management of this complex neurological disorder. Scalp-recorded EEG localization data are always used; however, it is postulated that visual EEG inspection alone may not always be sufficient to provide adequate sublobar localization, especially when invasive EEG studies are planned (5). However, the integration of ESI into clinical practice across epilepsy surgery centers varies, often reflecting the expertise and human resources available within each institution (6).

Recent studies exploring ESI, particularly from interictal low-density and high-density EEG recordings, demonstrated significant concordance with surgical resection areas and subsequent seizure-free outcomes (7–10). While visual EEG analyses are traditionally perceived as time-consuming, the advent of automated approaches presents a potential paradigm shift, offering the prospect of improved accuracy and speed, particularly when applied in a semi-automatic fashion within clinical contexts by experienced epileptologists (7).

Moreover, retrospective validation studies have underscored the high accuracy of ictal ESI, further bolstering its clinical utility (11). A notable prospective study involving 82 consecutive patients revealed that interictal ESI conferred additive value in approximately one-third of cases of drug-resistant focal epilepsy, highlighting its potential as a complementary tool in guiding therapeutic decision-making (12).

Despite these advancements, the absence of clear guidelines for clinical implementation of automated ESI analyses underscores the need for cautious interpretation and recognition of the limitations and pitfalls associated with fully automated methods. As the field continues to evolve, understanding the nuances of ESI and its optimal integration into the management algorithm of epilepsy patients remains an ongoing area of research and clinical consideration.

We aimed to assess the clinical utility of the implementation of semi-automatic ESI analyses as routine practice in consecutive patients with focal onset epilepsy undergoing presurgical evaluation.

## Methods

### Patients

**Figure 1:**
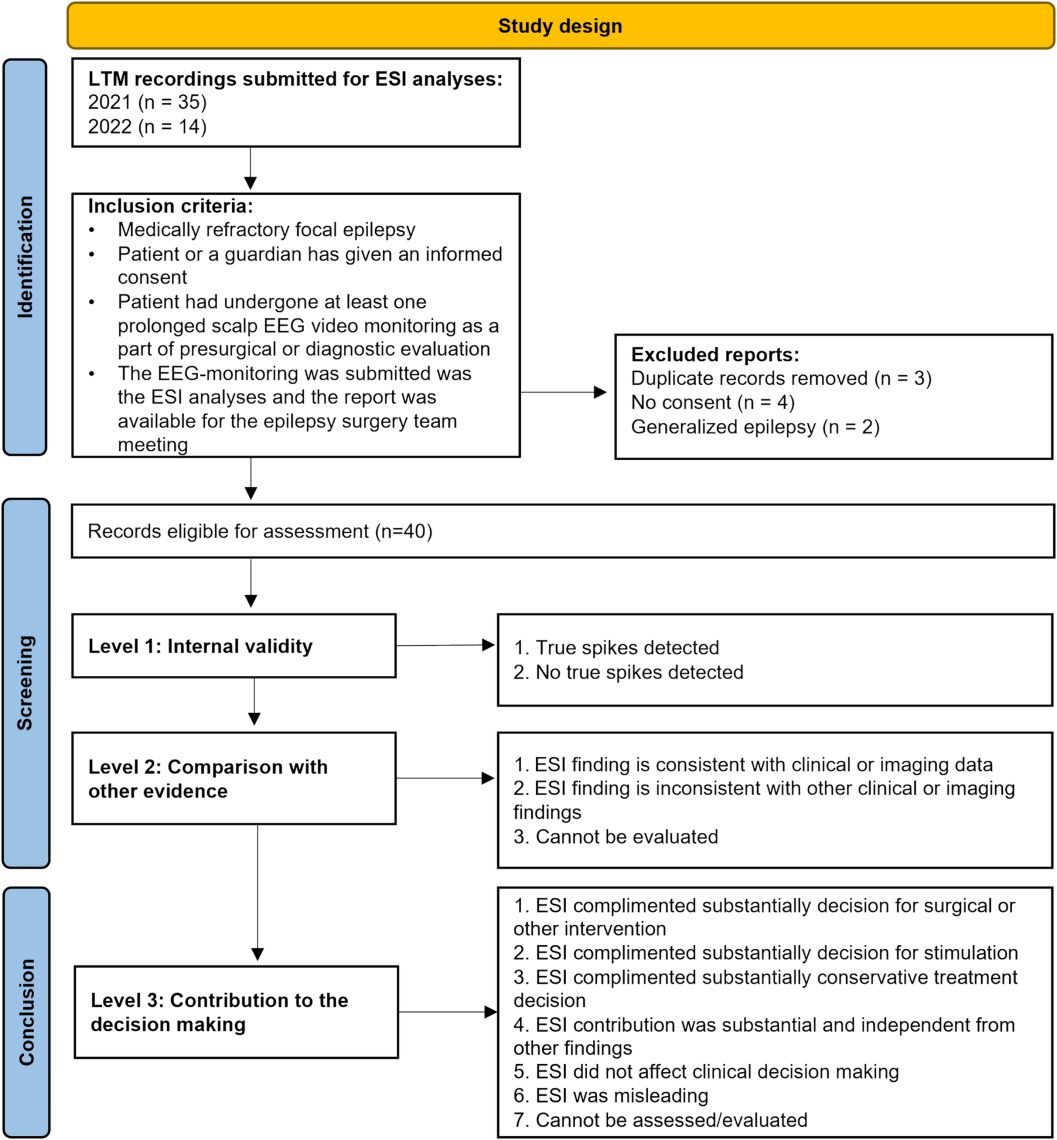
Study design and patients flowchart.

The study design and criteria for inclusion and evaluation are summarized in Figure 1. This study is part of a large ongoing study aimed at identifying clinically meaningful biomarkers in epilepsy patients. The studies involving human participants were reviewed and approved by the Regional Medical Research Ethics Committee of the Eastern Finland Collaborative Area. Written informed consent to participate in the study was obtained from the patient or the patient’s legal guardian. The principles of the Declaration of Helsinki were followed.

Forty-nine consecutive long-term EEG monitorings (LTM) were submitted for the automatic ESI analyses during 2021–2022. Of those, 40 presurgical patients fulfilled the following inclusion criteria: (i) medically refractory focal epilepsy, (ii) patient or guardian has given informed consent, (iii) patient underwent at least one prolonged scalp EEG video monitoring as part of presurgical evaluation, (iv) the EEG monitoring was submitted to the ESI analyses, and the report was available for the multidisciplinary team (MDT) meeting.

For each patient, the following demographic and presurgical evaluation data were collected: sex, age at LTM, age at first epileptic seizure, epilepsy duration, seizure type classified in accordance with the last ILAE recommendation for seizure classification, findings on magnetic resonance imaging (MRI) and 18F-fluorodeoxyglucose (18FDG-PET) imaging, and the conclusions of the MDT meeting.

### Long-term video-EEG monitoring (LTM) and automated ESI

All LTMs were carried out at the KUH Epilepsy Center. EEG-electrode setup was customized based on previous clinical knowledge of seizure semiology. For all except one patient, 37–41 electrodes were applied to the scalp according to the 10–20 system, with the addition of electrodes according to the 10–10 system depending on the individual EZ hypothesis (list of the electrodes Supplementary file 1). EEG recorded during LTM was analyzed using Clouds of Care services (Ghent, Belgium). MRI images were uploaded along with EEG files to the internet portal of Epilog. The detailed automated ESI pipeline (Epilog PreOp) was previously described (7). The reports obtained from the Epilog PreOp were presented at the MDT meeting. Automatically detected spike clusters were evaluated, and the source localization at the half-rising phase of the peak of the averaged spikes was used for the ESI localization on the lobar level.

### Imaging studies

All patients underwent high-resolution 3 T MRI scanning using an established protocol tailored to epilepsy patients. MRI was defined as “non-lesional” if no lesion relevant to the patient’s epilepsy had been detected by a certified neuroradiologist with expertise in epilepsy workup. After the ESI results were presented, the neuroradiologist re-evaluated MRI results; the final interpretation was used to classify MRI findings. Based on the localization of the MRI findings, patients were categorized into temporal lobe (TLE), extratemporal (ETLE), and extended (TLE + ETLE) groups. The group with extended MRI abnormalities included patients with multifocal MRI findings in different lobes or those who had extensive structural abnormalities extending from temporal to other lobes. 18FDG-PET was done if the MDT assessed it to be necessary for pre-surgical evaluation.

### Evaluation of contribution of ESI results to the clinical decision

The authors (two adult neurologists, a pediatric neurologist, a clinical researcher, two clinical neurophysiologists, and a neuroradiologist) evaluated the contribution of the ESI results in relation to clinical decisions made by the MDT after phase 1 investigations and classified the utility of the ESI results for the decisions made by the MDT at that time point. The authors were the core members of the KUH Epilepsy Center’s MDT and had participated in the decision-making throughout the process. The classification categories were summarized in Figure 1. Other intervention involved selecting the invasive stereo-EEG study.

### Statistical analyses

Statistical analyses were performed with the IBM Statistical Package for the Social Sciences (SPSS) version 29 (SPSS Inc., Chicago, IL, USA). The Pearson Chi-Square test was used to assess the association between ESI consistency and ESI clinical utility for the clinical decision in the whole cohort and patient groups stratified based on treatment choice (surgical vs. other treatments) and type of MRI lesion. A *p*-value <0.05 was considered significant. Results were presented as mean ± standard deviation (SD) or number and percentage of patients within the studied group.

## Results

In this group of 40 patients (27 female), the mean age was 35.0 years (±12.0). The mean age at onset of the first seizure was 18.1 years (±12.3), and the duration of epilepsy was 16.0 years (±13.3). The cohort included two pediatric patients (age at admission < 18 years).

Based on MRI findings, 20 patients were categorized as lesional before the presentation of ESI results, while in the other 20 patients, no evident lesion was found even after tailoring MRI inspection to ESI results. FDG-PET was performed in 36 out of 40 patients (90%), and positive local hypometabolism findings were observed in 22 (61%) of these patients. A summary of MRI findings and epileptic seizure types relevant to clinical decision-making was presented in Table 1. Focal onset impaired awareness seizures, either manifesting with automatisms or cognitive symptoms, were the most common in our study population.

**Table 1:** Clinical, imaging and ESI findings.

Clinical, imaging, ESI findings and MDT decision	All patients (*n* = 40)	Other treatments group (*n* = 30)	Resective surgery group (*n* = 10)
Age	35 ± 12 (2–59)	33.5 ± 12.9 (2–59)	39.3 ± 8.1 (29–53)
Sex (Male/Female)	13/27	21/7	6/4
Age at onset	18.2 ± 123 (0–50)	16.8 ± 11 (0–36)	22.3 ± 15.5 (3–50)
Epilepsy duration before LTM	16.4 ± 12.2 (2–47)	16.4 ± 11.6 (2–47)	16.5 ± 14.7 (3–42)
Seizure type			
2.0 Focal seizure—Unspecified	2 (5%)	2 (6.7%)	0
2.1.2.3 Focal onset seizure without impaired awareness—Nonmotor onset—Cognitive	1 (2.5%)	1 (3.3%)	0
2.1.2.5 Focal onset seizure without impaired awareness—Nonmotor onset—Sensory	1 (2.5%)	1 (3.3%)	0
2.2.1 Focal onset impaired awareness seizure—Motor onset	4 (10%)	3 (10%)	1 (10%)
2.2.1.1 Focal onset impaired awareness seizure—Motor onset—Automatisms	7 (17.5%)	6 (20%)	1 (10%)
2.2.2 Focal onset impaired awareness seizure—Nonmotor onset	2 (5%)	2 (6.7%)	0
2.2.2.1 Focal onset impaired awareness seizure—Nonmotor onset—Autonomic	2 (5%)	1 (3.3%)	1 (10%)
2.2.2.2 Focal onset impaired awareness seizure—Nonmotor onset—Behavior arrest	6 (15%)	4 (13.3%)	2 (20%)
2.2.2.3 Focal onset impaired awareness seizure—Nonmotor onset—Cognitive	8 (20%)	4 (13.3%)	4 (40%)
2.2.2.4 Focal onset impaired awareness seizure—Nonmotor onset—Emotional	2 (5%)	2 (6.7%)	0
2.2.2.5 Focal onset impaired awareness seizure—Nonmotor onset—Sensory	4 (10%)	3 (10%)	1 (10%)
2.3 Focal to bilateral tonic–clonic	1 (2.5%)	1 (3.3%)	0
MRI findings			
MRI non-lesional	20 (50%)	20 (66.7%)	0 (0%)
MRI lesional	20 (50%)	10 (33.3%)	10 (100%)
TLE	10 (50%)	3 (30%)	7 (70%)
ETLE	3 (15%)	3 (30%)	0 (%)
TLE + ETLE	7 (35%)	4 (40%)	3 (30%)
Type of the MRI lesion (*n* = 20)			
Focal cortical dysplasia (FCD)	4 (20%)	1	3
Hippocampal sclerosis (HS)	3 (15%)	1	2
Dysembryoplastic neuroepithelial tumors (DNET)	1 (5%)	0	1
Encephalocele	2 (10%)	1	1
Cavernoma	2 (10%)	0	2
Heterotopia	2 (10%)	2	0
Tuberous sclerosis	1 (5%)	1	0
Previous surgical resection	2 (10%)	2	0
Other*	2 (10%)	2	0
Hippocampal sclerosis (HS) and Focal cortical dysplasia (FCD)	1 (5%)	0	1
ESI findings			
LTM duration (h)	94.2 ± 37.0 (18–169.5)	91.1 ± 39.4 (18–166.8)	103.4 ± 17.5 (77.4–169.5)
Number of spikes of the largest cluster	2,460 ± 2,946 (30–12,420)	1,736 ± 2,337 (30–8,630)	4,341 ± 3,622 (617–12,420)
Hemispheric ESI localization			
Right	5 (12.5%)	4 (13.3%)	1 (10%)
Left	8 (20%)	4 (13.3%)	4 (40%)
Independent bilateral	23 (57.5%)	18 (60%)	5 (50%)
No spikes	4 (10%)	4 (13.3%)	0 (0%)
Lobar ESI localization			
Temporal	23 (57.5%)	16 (53.3%)	7 (70%)
Frontal	9 (22.5%)	8 (26.7%)	1 (10%)
Occipital	1 (2.5%)	0 (0%)	1 (10%)
Insula	3 (7.5%)	2 (6.7%)	1 (10%)
No spikes	4 (10%)	4 (13.3%)	0 (0%)
ESI consistency			
ESI finding is consistent with clinical or imaging findings	28 (70%)	20 (66.7%)	8 (80%)
ESI finding is inconsistent with other clinical or imaging findings	7 (17.5%)	5 (16.7%)	2 (20%)
Cannot be evaluated	5 (12.5%)	5 (16.7%)	0 (0%)
MDT decision			
Surgical resection	8 (20%)	NA	8 (80%)
Invasive evaluation	8 (20%)	6 (20%)	2 (20%)
Stimulation	3 (7.5%)	3 (10%)	0 (0%)
Non-surgical treatment	14 (35%)	14 (46,7%)	0 (0%)
Pending decision	5 (12.5%)	5 (16.7%)	0 (0%)
Discontinued/patient seizure free	1 (2.5%)	1 (3.3%)	0 (0%)
New LTM	1 (2.5%)	1 (3.3%)	0 (0%)

ESI, electrical source imaging; LTM, long-term monitoring; TLE, temporal lobe epilepsy; ETLE, extratemporal lobe epilepsy; MDT, multidisciplinary team. * Craniopharyngioma, post-hemorrhage atrophy.

On average, the duration of LTM recorded EEG submitted for ESI analysis was 94.2 h (±36.0 h). Overall, automatic analyses identified true interictal epileptiform discharges (IEDs) in 36 LTM recordings. In the remaining four cases, automatic analyses did not identify IEDs in two patients, and in two other patients, identified IEDs were classified as physiological rhythms and artifacts upon review. In these four cases, ESI consistency with other findings could not be evaluated. The number of automatically identified IED clusters was 3.7 (±0.8); after review by a clinical neurophysiologist before the MDT meeting, the ESI-detected IED clusters decreased to 2.9 (±1.1). Bilateral IED clusters were observed in 23 patients, with the most common localization being the temporal lobe (*n* = 23). A full summary was provided in Table 1. ESI findings were classified as consistent with clinical or imaging findings in 28 out of 40 patients (70%).

During the first stage of pre-surgical evaluation, a resective surgical procedure or invasive exploration was suggested for 16 patients, and 10 of these patients underwent surgery. After a one-year follow-up, an Engel IA or IB outcome was reported in 7 out of 10 patients.

**Figure 2:**
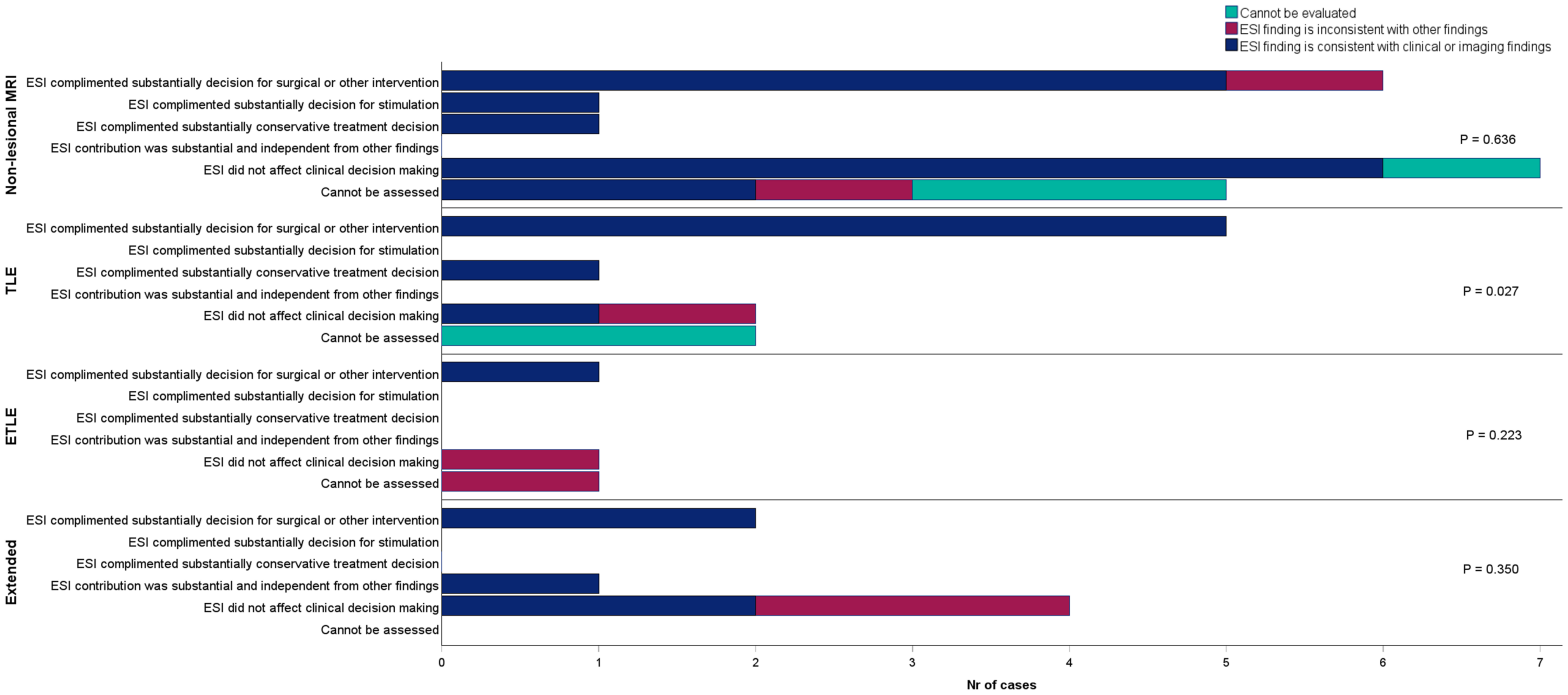
Association between ESI consistency and ESI utility for the decision-making process in patients stratified by MRI findings. ESI, electrical source imaging; TLE, temporal lobe epilepsy; ETLE, extratemporal lobe epilepsy.

In the entire study population, ESI results substantially complemented either the surgical or conservative treatment choice in 45% of cases. However, ESI findings were classified as a substantial independent factor for clinical decision-making in only one case. In this case, the ESI results strongly affected the choice between invasive exploration and conservative treatment. Also, the ESI results significantly modified the planning of the stereo-EEG study. A brief case description if provided in Supplementary file 2. ESI did not significantly affect clinical decision-making in 35% (14 out of 40) of the cases. In 20% of cases, including those with no spikes, pending decisions, or insufficient other information to form a hypothesis, the value of ESI could not be evaluated (Table 2). Overall, the impact of ESI results on clinical decisions did not reach statistical significance in the whole population (Pearson chi-square *p* = 0.05). In the subgroup of patients who underwent resective surgery, ESI results consistent with clinical and imaging findings were significantly more commonly associated with the concordant clinical decision (Pearson chi-square *p* = 0.016). Furthermore, the clinical utility of ESI findings was more consistent in the group of patients with temporal lobe MRI lesions (Figure 2, Pearson chi-square *p* = 0.027).

**Table 2:** Association between ESI consistency and ESI utility for the decision-making process in the whole cohort and resective surgery group.

Other treatments group (*N* = 30)	ESI finding is consistent with clinical or imaging data (*N*)	ESI finding is inconsistent with other clinical or imaging findings (*N*)	Cannot be evaluated (*N*)	Total (*N* (%))	Pearson Chi-Square *p*-value
ESI complimented substantially decision for surgical or other intervention	6	1	0	7 (23.3%)	
ESI complimented substantially decision for stimulation	1	0	0	1 (3.3%)	
ESI complimented substantially conservative treatment decision	2	0	0	2 (6.7%)	
ESI contribution was substantial and independent from other findings	1	0	0	1 (3.3%)	
ESI did not affect clinical decision making	8	2	1	11 (36.7%)	
ESI was misleading	0	0	0	0 (0%)	
Cannot be assessed	2	2	4	8 (26.7%)	0.300
Resective surgery group (*N* = 10)					
ESI complimented substantially decision for surgical or other intervention	7	0	0	7 (70%)	
ESI complimented substantially decision for stimulation	0	0	0	0 (0%)	
ESI complimented substantially conservative treatment decision	0	0	0	0 (0%)	
ESI contribution was substantial and independent from other findings	0	0	0	0 (0%)	
ESI did not affect clinical decision making	1	2	0	3 (30%)	
ESI was misleading	0	0	0	0 (0%)	
Cannot be assessed	0	0	0	0 (0%)	0.016
Whole cohort					
ESI complimented substantially decision for surgical or other intervention	13	1	0	14 (35%)	
ESI complimented substantially decision for stimulation	1	0	0	1 (2.5%)	
ESI complimented substantially conservative treatment decision	2	0	0	2 (5.0%)	
ESI contribution was substantial and independent from other findings	1	0	0	1 (2.5%)	
ESI did not affect clinical decision making	9	4	1	14 (35%)	
ESI was misleading	0	0	0	0 (0%)	
Cannot be assessed	2	2	4	8 (20%)	0.050
Total	28	7	5	40 (100%)	

ESI, electrical source imaging; *N*, number.

## Discussion

Our study contributes to the evolving field of epilepsy surgery by evaluating the clinical utility of semi-automated ESI in the pre-surgical assessment of patients with drug-resistant epilepsy. The integration of ESI may aim not only to assist in surgical decision-making but also to guide the selection of conservative treatment options, reflecting a broader spectrum of clinical applications beyond traditional approaches.

In our cohort, ESI findings were consistent with outcomes concluded in MDT meetings for a significant majority (70%) of patients. Notably, in almost half of the cases (45%), ESI provided additional insights that influenced treatment decisions, with the most substantial impact observed in determining the suitability for surgical or invasive exploratory procedures. However, it is important to note that MDT considered the results of ESI analyses as having no impact on the clinical decision in 35% of this cohort. The significant additive value was seen in the subpopulation of patients who underwent resective surgical procedures and in those with MRI lesions in their temporal lobes. Nevertheless, independent decision-making solely based on ESI findings was achieved in only one patient, suggesting that ESI played a largely supplementary role in the clinical workflow. Previous ESI studies, including meta-analyses, showed some tendency for ESI being concordant with the resection extension and good outcome in TLE. However, the findings were rather heterogeneous in other studies (9, 13–16). High concordance results between ESI and clinical outcomes were also reported in MRI-negative patients and patients with malformations of cortical developments (MCDs) (9, 16–19). We failed to see the association between ESI results and clinical decision in patients with lesions outside the temporal lobe (*n* = 5) or non-lesional cases (*n* = 20). In our cohort, although MRI scans were re-evaluated after the ESI findings were presented to the MDT, the number of MRI-positive cases remained unchanged. Thus, in our study, ESI did not enhance the diagnostic yield of MRI evaluations. Our study cohort also included only five patients with MCDs, thus not allowing us to draw further conclusions specific to this group.

Overall, in cases with systematically concordant data, the addition of ESI—by further strengthening the chain of evidence—is likely to be perceived positively. However, ESI cannot be reliably utilized when no interictal spikes are recorded or when spikes cannot be clearly identified for analysis. Moreover, in our cohort, some ESI results obtained through automated analyses were discordant with the known MRI lesion or the clinical semiology (17% of the cases). Among the discordant cases were two MRI-negative patients, one of whom had a very low spike count, limiting the reliability of source localization. Discordance was also observed in MRI-positive cases involving parietal MCD, a temporal cavernoma, extensive periventricular heterotopia, prior surgical resection, and a craniopharyngioma. It is expected that deep or extensive lesions may not yield precise ESI colocalization. This reflects a known limitation of ESI in accurately localizing epileptiform activity arising from complex or deep-seated lesions. Additionally, cavernomas can distort the surrounding cortical architecture and alter the propagation of epileptiform discharges and seizure onset in these cases may lie in the perilesional cortex rather than the lesion itself, further complicating the interpretation of ESI findings.

Previous studies have predominantly focused on surgically treated cohorts, demonstrating high-density (hdESI) and low-density (ldESI) ESI’s diagnostic sensitivity and specificity for EZ, validated through postoperative outcome or concordance with intracranial recordings (10, 15, 17, 20–22). Systematic reviews and meta-analyses have further consolidated these findings, confirming ESI’s additive diagnostic value in approximately one-third of pre-surgical cases (12, 14, 18, 23). Our study was underpowered to explore the additive value of ESI results in comparison to surgery extent and surgical outcome.

The practical feasibility of implementing ESI in routine clinical practice is a crucial consideration (24). In our experience, the implementation of automated spike detection and source localization using the commercial platform proved highly effective, identifying true epileptic spikes in 90% of consecutive presurgical patients. Studies evaluating the time and resource requirements have indicated substantial initial setup investments, with subsequent reductions in physician time following implementation (25). To our knowledge, there are no studies directly evaluating the cost-effectiveness of different ESI workflows or analysis pipelines. There are several free, open-source, and commercial software packages available, which can be chosen based on the financial and human resources of the center (24–26). However, the licensing of software for clinical use may be a restrictive factor for some solutions. A recent study by Reus et al. (27) identified 14 barriers and 14 enablers for the future implementation of automatic spike detection, highlighting the need for further software development to increase users’ trust and efficiency in EEG analysis workflows. Additionally, some epilepsy centers are reluctant to adopt ESI due to perceived limited additional benefits beyond conventional diagnostic methods (25).

Recent advancements in hdESI have shown promising results in the semi-automatic detection of IEDs, demonstrating accuracy comparable to visual analysis in delineating resection zones (28, 29). Moreover, several studies have indicated that long-term low-density EEG recordings with 25–37 electrodes may be sufficient to obtain clinically reliable ESI results (9, 25). The study by Spinelli et al. (9) suggested that semi-automatic ESI analyses might overcome the limitations of low-density EEG by efficiently increasing the number of identified IEDs for ESI analyses, thus mitigating the signal-to-noise ratio problems often seen with fewer IEDs in shorter recordings. It has also been noted that it is common to find two or more IED subtypes in the EEG, and modeling these subtypes might be crucial for correct EZ delineation (30, 31).

The advantage of the automatic method we implemented in our work-up was that it provided a comprehensive summary of up to four automatically identified IED clusters without significantly impacting the time needed by a clinician to interpret the results within the clinical context. The time-related efficiency of this automated platform was previously highlighted in one study (25). However, our study further emphasizes the importance of visually evaluating the ESI-identified IED clusters. We found that automatically reported IEDs were not true in two patients, and the number of relevant clusters decreased after the results were reviewed by an experienced clinical neurophysiologist. Moreover, 64% of patients exhibited independent ESI clusters bilaterally. This underscores the necessity of integrating ESI results with comprehensive clinical and imaging assessments to accurately ascertain the EZ.

There is also growing evidence that modeling the early phases of the IED is more reliable compared to commonly used half-rise modeling (10, 15, 22, 32). We used source localization at the half-rising phase of the peak for our clinical decision-making during MDT meetings, based on the more commonly accepted practice to improve the signal-to-noise ratio. Additionally, the maps of the single IEDs provided in the report were considered by the MDT. When the ESI analysis was first implemented into the clinical workup, we did not include manual marking of IEDs for the analysis. It was suggested that, in addition to manually marked spikes, Epilog’s automatic platform could detect additional spike clusters and thus potentially increase the benefit of ESI for clinical decision-making (25).

Our study is constrained by a relatively small sample size, especially of surgically treated patients, which precludes robust analyses of the correlation between ESI findings and surgical outcomes. This also warrants caution when evaluating the impact of ESI in smaller subgroups of patients, such as those with ETLE or extended lesion findings. The ictal ESI analyses were done for only three patients; therefore, their findings were not evaluated with respect to clinical decisions. Although the results of automatic ESI were evaluated, as advised in the clinical context, by a panel of experienced epileptologists and neurophysiologists, one can argue that subtle findings might have been missed during the automatic ESI analyses. The evaluation of the automatic results requires substantial experience in interpreting EEG findings, as only 20 representative IEDs are presented in the reports used by our center. We consider it an advantage of our study that automatic ESI reports were reviewed and presented to the MDT by clinical neurophysiologists familiar with the patients’ LTM results.

In conclusion, this study presents a single-center experience in implementing commercially available semi-automatic ESI analyses into the pre-surgical evaluation protocol for drug-resistant patients with focal epilepsy. Our results demonstrate good concordance and support the clinical utility of semi-automated ESI in enhancing the precision of pre-surgical evaluation. However, we consider the impact of the ESI results to be supplementary for the majority of patients undergoing pre-surgical work-up. Additionally, in about one-third of the patients, ESI results are likely to have no impact on clinical decision-making. Nevertheless, we also conclude that the highest clinical value is seen in patients who can receive resective surgery. The findings underscore the importance of integrating ESI with other diagnostic tools and clinical assessments to optimize patient outcomes.

## Data Availability

The raw data supporting the conclusions of this article will be made available by the authors without undue reservation.
