# Supplementary material for: Clinical utility of semi–automated EEG electric source imaging of interictal discharges in presurgical evaluation and surgical treatment decision making
Source: Front Neurol. 2025 Sep 3;16:1598265. doi: 10.3389/fneur.2025.1598265 (PMC12440761; doi:10.3389/fneur.2025.1598265)
Supplement: Supplementary file 1 [file Data_Sheet_1.pdf]

Supplementary file 1. List of EEG Electrodes Included in the LTM Montages

The standard 10–20 system electrodes were included in all long-term monitoring (LTM) studies. Additional 10–10 system electrodes were selected based on the individual epileptogenic zone (EZ) hypothesis, allowing for denser coverage of the frontal and centro-parietal regions or the parietal and occipital regions as clinically indicated.

10–20 System electrodes included in all LTMS

Fp1, Fp2

F7, F8

F3, F4

C3, C4

T3, T4

T5, T6

P3, P4

O1, O2

Fz, Cz, Pz

10–10 System electrodes included depending on the hypothesis

FT9, FT10 (in all LTMs)

FT11, FT12

F5, F6

FC1, FC2, FC3, FC4, FC5, FC6

C1, C2, C5, C6

CP1, CP2, CP3, CP4, CP5, CP6

TP7, TP8

P5, P6

PO3, PO4

CPz, POz, Oz
