# Supplementary material for: Clinical utility of semi–automated EEG electric source imaging of interictal discharges in presurgical evaluation and surgical treatment decision making
Source: Front Neurol. 2025 Sep 3;16:1598265. doi: 10.3389/fneur.2025.1598265 (PMC12440761; doi:10.3389/fneur.2025.1598265)
Supplement: Supplementary file 2 [file Data_Sheet_2.pdf]

Supplementary file 2. Case description

The patient is a 28-year-old individual with no relevant family history of epilepsy. Focal-onset epilepsy was diagnosed at the age of 13 years. Brain MRI demonstrated post-hemorrhagic atrophy involving the right temporal and occipital lobes. During long-term video-EEG monitoring (LTM), seizures with impaired awareness non-motor onset (behavioral arrest) were recorded. The ictal onset was localized to the right posterior temporal region based on scalp EEG findings.

Automated electrical source imaging (ESI) identified spike clusters over electrodes FT9 and T6, with detailed review focusing on T6. Given the extent of the MRI-documented lesion, the ESI findings were deemed the most informative electrophysiological data. These results significantly influenced both the clinical decision to proceed with stereoelectroencephalography (SEEG) and the strategic planning of electrode placement.
